# Evaluation of cropping method for perennial ratoon rice: Adaptation of SALIBU to triple-cropping in Vietnam

**DOI:** 10.12688/f1000research.20890.3

**Published:** 2020-09-30

**Authors:** Masato Oda, Huu Chiem Nguyen, Van Thao Huynh

**Affiliations:** 1 Crop, Livestock and Environment Division, Japan International Research Center for Agricultural Sciences, Tsukuba, 305-8686, Japan; 2 Environmental Sciences, Can Tho University, Can Tho, Vietnam

**Keywords:** The Mekong Delta, Triple rice cropping, Methane mitigation, Row input, Sustainable, Taguchi method, Effect size

## Abstract

**Background**: Generally, the yield of ratoon rice is at most 50% of the main crop. However, a cropping method “SALIBU” achieved more yield than the main crop and enables the perennial cropping. Although the SALIBU method is implementing 10 additional management practices to conventional method in Indonesia, the effect of each management practice is unclear.

**Methodology**: We evaluated the effect size using an L _16_ orthogonal array design pot experiment in triple-cropping rice in Vietnam. The robustness was checked by duplicating the experiment under standard and poor conditions.

**Results and Discussion**: Positive large effects were shown in the poor conditions only.  Cutting twice most affected the number of ratoon tillers. Importantly, the effect was positive under poor conditions but negative under standard conditions. Late irrigation had a robust negative effect. No treatment is effective in the triple-cropping of standard conditions. The SALIBU includes practices with unstable, negative, or minimal effects. The unstable effects show the interaction with the condition. The practices that have negative effects should exclude. Using practice on small effect size should depend on a cost-benefit analysis.

**Conclusions**: No additional practice is effective for changing the triple-cropping to perennial ratoon cropping except harvesting near the ground. However, further work will be conducted to clarify the interaction between cutting twice and the cultivation condition.

## Introduction

### Ratoon rice

Rice is usually an annual crop but can be renewed using the ratoon cropping method. Perennial cropping of rice requires less labor and water, while reducing climate risk and greenhouse gas emissions. Perennial rice cropping is a traditional cropping method but is rarely used because of the low yield. However, the additional yield achieved after harvesting the main crop has allowed its successful commercial application in the southern region of the United States of America and parts of southern China ( Sacks, 2013). Most studies on ratoon rice have focused on additional yield, and the yield is at most 50% of the main crop ( Negalur *et al.*, 2017). A previous study reported a yield of up to 90% of the main crop using a special variety (PR23), but the fluctuations in yield were very large ( Zhang *et al.*, 2014).

### New approach using lower node

Recently, a breakthrough in increasing yield was achieved in Indonesia. Rice has limited growth period during winter; therefore, rapid growth is the key to success of ratoon cropping. Moreover, ratoon cropping from higher nodes of rice is important, because the carbon can be accessible to the main crop culm ( Balasubramanian *et al.*, 1992). However, because tropical regions have no winter, the method used can be different. Fitri *et al.* (2019) looked at an updated method of traditional perennial rice cropping developed by Mr. Erdiman in 2010. The method was named “SALIBU,” a portmanteau of the Indonesian words “SALIN” (replication) and “IBU” (mother). Using SALIBU, the same or higher yield than that of the main crop was achieved. The mechanisms inducing the high yield have not been well studied, but a possible reason is that lower nodes can extend new roots and improve nutrient uptake from the soil ( Yamaoka *et al.*, 2017). The implementation of SALIBU has been successful in different areas and at different elevations and groundwater levels in Indonesia ( Fitri *et al.*, 2019), and has recently spread to Myanmar ( Yamaoka *et al.*, 2017).

### Evaluation of management practices

However, even if the similar conditions, in some cases, the yield is less than 50% of the main crop yield ( Pasaribu *et al.*, 2018). This means the robustness of SALIBU cropping method is not enough. To adapt the SALIBU cropping method to areas with different growing conditions, we should evaluate the performance of each management practice, and modify the practices to suit the conditions. We aimed to adapt the SALIBU method to direct seeding triple-cropping of rice in the Mekong Delta, so that we evaluated the effect size of each practice and that robustness. Here, we found that the most effective (positive) management practice under poor conditions had an adverse effect (negative) under standard conditions. Furthermore, we found that the practice has a robust negative effect on the yields under both poor and standard conditions.

## Methods

Evaluating each practice of a cropping method under different conditions is difficult because of the huge number of possible combinations. We have summarized the management practices of SALIBU method ( Yamaoka *et al.*, 2017) into four practices. We allocated those practices to two levels of an L _16_ orthogonal array ( Taguchi, 1986) and conducted a pot experiment. The Taguchi method is a popular method to test the robustness of technologies in actual condition by artificial condition. To test robustness, the experiment was replicated under standard conditions and poor conditions, namely low plant density, no fertilization, continuous flood water management, and late harvesting: these conditions are known to reduce the yield of ratoon cropping of rice ( Negalur *et al.*, 2017). We analyzed the effect of each of the four practices on ratoon tillers and yield. Then, we evaluated the robustness of the effect of practices between the two conditions.

### Materials

The pot experiment was conducted in a fine net house at Can Tho University (Can Tho city, Vietnam) from December 2018 to June 2019. We used 38 cm × 58 cm wide and 30 cm high containers. All containers were filled up to 20 cm with paddy soil. The soil was collected from topsoil (about 25 cm) of a paddy field at TL2 Hamlet, Thuan Hung village, Thot Not district, Can Tho city, Vietnam, just after natural flooding of the Mekong River and used on the day it was collected. The soil was well mixed in advance. Germinated seeds (Jasmin 85 variety from Can Tho University, popular in the Mekong Delta) were used. Jasmin is an Indica and has characteristics unsuitable for ratoon cropping of rice ( Negalur *et al.*, 2017). These disadvantages will amplify the effects of the practices. We used urea (46% N), single superphosphate (16% P _2_O _5_), and potassium chloride (61% K _2_O) as fertlizers; the applied amount of those contents (kg ha ^-1^) used for each treatment are given in the following section.

### Treatments

SALIBU management consists of cutting near the ground and nine special management practices in addition to the conventional cropping management practice of rice transplanting ( Yamaoka *et al.*, 2017). The practice of early harvesting (physiological maturity; 25% green color husk) is conventional in Mekong Delta triple-cropping cultivation. The rest of the practices are as follows. (1) Pre-fertilization: 25 kg ha ^−1^ N and 46.75 kg ha ^−1^ P _2_O _5_ at seven days before harvesting. (2) Cutting twice: all rice was harvested 25 cm above the ground, then cut again beneath the first node above ground on day seven (or day zero for control plants) after harvesting (rice straws were returned to the ground). The recommendation is to cut 3–5 cm above ground; we kept only the node below ground. (3) Late irrigation: irrigation was started on day 14 (or day seven for control plants) after harvesting (the water table was about 5 cm until irrigation started). (4) Adjusting: the practice consisted of (a) hand weeding, (b) dividing hills into two or three tillers and replanting to fill the space, (c) pushing the rice plants into the soil if the root came up on soil surface, (d) removing excess plants to keep original plant density, and (e) draining from day 29 to 43 after harvesting (though (e) is not “adjusting”, it is technically inseparable because “adjusting” requires draining). We did the pot experiment using an L _16_ orthogonal array design ( Oda *et al.*, 2019). We set the pots randomly in the fine net house.

### Conditions

The standard conditions were based on the standard of direct seeding triple-cropping rice in the Mekong Delta: the plant density was 230 kg ha ^−1^ dry weight (about 173 seeds per pot), fertilizer was applied three times on day seven (27.6 kg ha ^-1^ N, 45.2 kg ha ^-1^ P _2_O _5_, 3.68 kg ha ^–1^ K _2_O), 20 (36.7 kg ha ^-1^ N), and 42 (27.6 kg ha ^-1^ N, 3.68 kg ha ^-1^ K _2_O) after seeding, with alternate wet and dry water management (15 to 5 cm; from seven days after seeding to 10 days before harvesting).

The poor conditions were as follows: low plant density (nine plants per pot), no fertilization (except the pre-fertilization treatment), continuous flooding water management (0 to 5 cm, from seven days after seeding to 10 days before harvesting), and late harvesting (seeded 10 days before the standard condition plants and harvesting on the same day of harvesting as the standard conditions). These conditions are known to negatively affect ratoon cropping of rice ( Negalur *et al.*, 2017).

### Analysis

We recorded the number of plants and ratoon tillers at the harvesting time. We immediately oven-dried the sample then weighed the grain and straw. We analyzed the effect of the practices using the mean value and Cohens’ *d* effect size using the following formula ( Cohen, 1992):

$$d=\frac{M_{1}-M_{2}}{\sqrt{\frac{SD_{1}^{2}+SD_{2}^{2}}{2}}}$$

where, *d* is the effect size, *M*_1_ is the mean of treatment, *M*_2_ is the mean of un-treatment, and SD is standard deviation.

The p value of the significance test is affected by the sample size and cannot be used to assess the effect. Measuring effect sizes allows for evaluation involving variance and is not affected by the sample size. Data were processed using Microsoft Excel 2016.

## Results and discussion

We examined the SALIBU management practices using an L _16_ orthogonal array design pot experiment and duplicated the experiment under standard and poor conditions. The ratoon rice yield was proportional to straw biomass, and the straw biomass was proportional to the number of ratoon tillers. Cutting twice had the highest effect, and the effect was reversed between the standard and poor conditions. Furthermore, late irrigation had a robust negative effect ( Oda *et al.*, 2019). The results of the effect size analysis show that the SALIBU cropping method includes practices that are unstable, negative, or small. Improving these practices could improve the method. Perennial ratoon rice cropping will be possible for the Mekong Delta triple-cropping rice with the sole practice of harvesting rice near the ground because positive large effects were shown in the poor condition only. This also means standard condition is robust.

### Yield component

**Figure 1.**
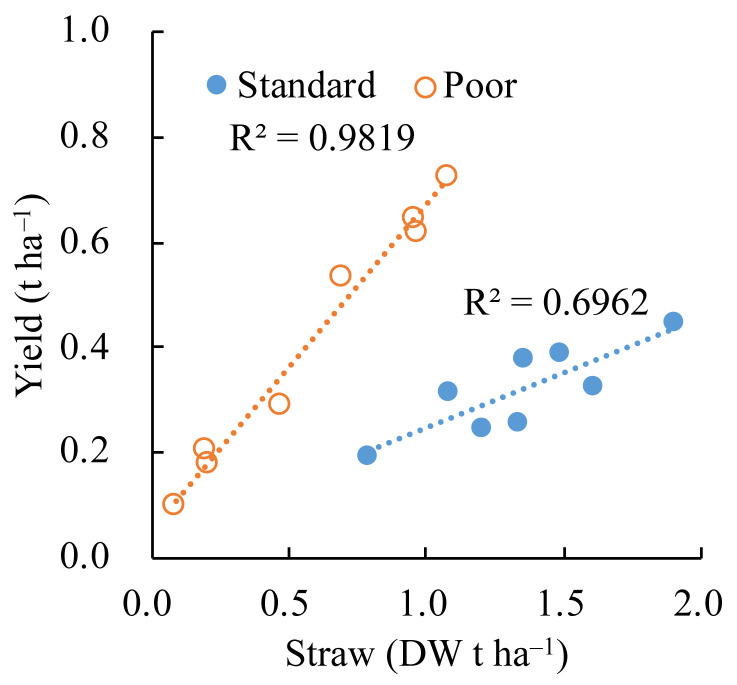
Straw biomass vs yield. DW, dry weight.

**Figure 2.**
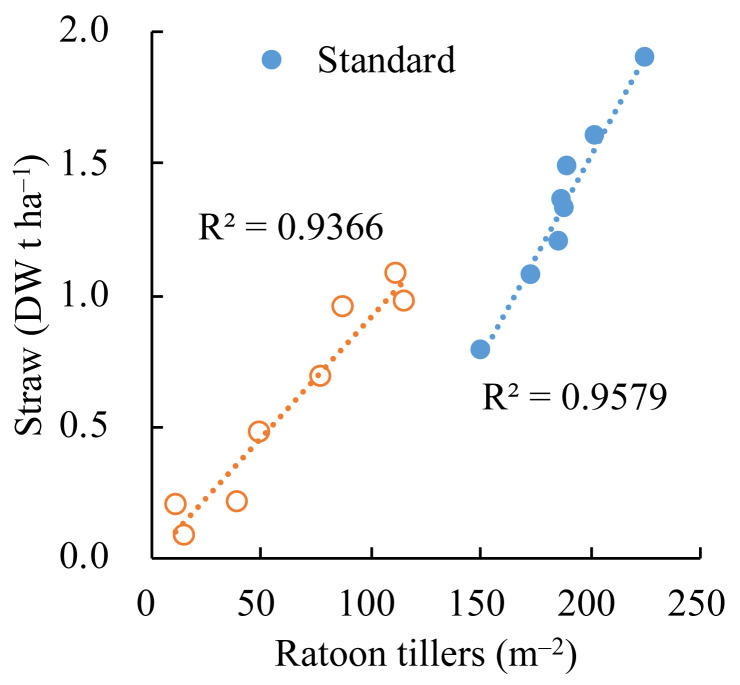
Ratoon tillers vs straw biomass.

**Figure 3.**
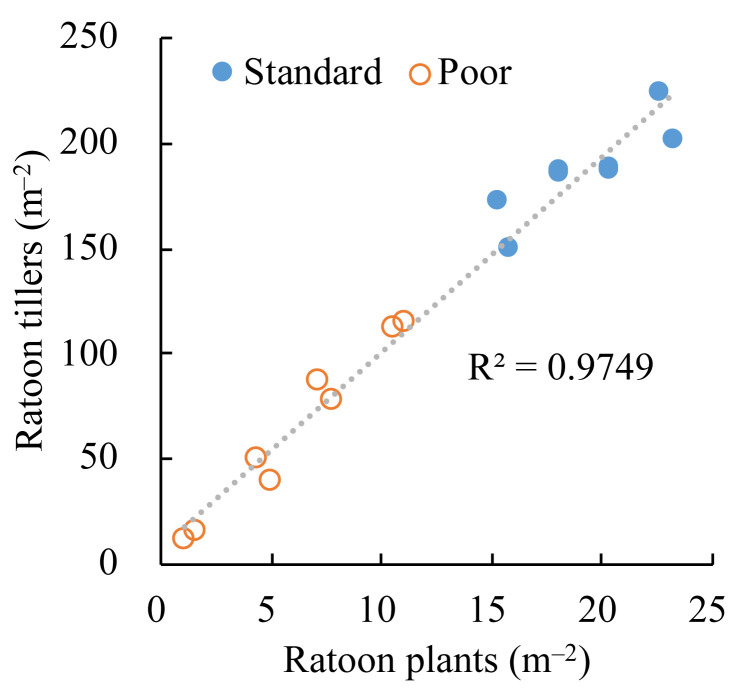
Ratoon plants vs Effective tillers.

The ratoon rice yield was proportional to straw biomass. The harvest index under poor conditions was higher than that under standard conditions ( Figure 1). Importantly, straw biomass was proportional to the number of ratoon tillers under both conditions ( Figure 2). The ratoon rice yield is determined by the number of ratoon tillers, and the relationship between the number of ratoon tillers and the yield is consistent with those reported in a previous study ( Pasaribu *et al.*, 2018). The number of ratoon tillers was also in proportion to the number of plants under poor conditions ( Figure 3), although it is important to note that under poor conditions, half of the pots had no ratoons.

### Effect of practices

We examined the effect of management practices such as pre-fertilization, cutting twice, late irrigation, and adjusting on the number of ratoon tillers.

Importantly, the effect of cutting twice was positive under poor conditions but was negative under standard conditions. In other words, there is an interaction between the practice and the condition. The average cutting heights (length of the first node) of the standard condition plants were 5.5 cm (cutting twice) and 4.0 cm (harvesting time), and those of the poor conditions were 6.8 cm and 3.0 cm, respectively. The extensions of nodes were smaller under standard conditions than those under poor conditions. There is no consensus about the ideal cutting height ( Negalur *et al.*, 2017), although previous studies were not carried out using the SALIBU method.

Furthermore, late irrigation had a negative effect on the number of ratoon tillers under both conditions ( Table 1). This might be a drawback of the pot experiment method due to decreased percolation; however, this is unlikely because the SALIBU method is successful in the lowlands ( Fitri *et al.*, 2019).

**Table 1.** Number of ratoon tillers by practice (m ^−2^).

Practice		Mean		s.d.	
		Standard	Poor	Standard	Poor
Pre- fertilization	+	187	112	151	110
	–	188	15	149	37
Cutting twice	+	150	116	147	109
	–	225	12	143	29
Late irrigation	+	186	40	102	68
	–	189	87	186	114
Adjusting	+	172	50	152	93
	–	202	78	147	99

The mean value of the practices, *n* = 8.

### Robustness of effects

**Figure 4.**
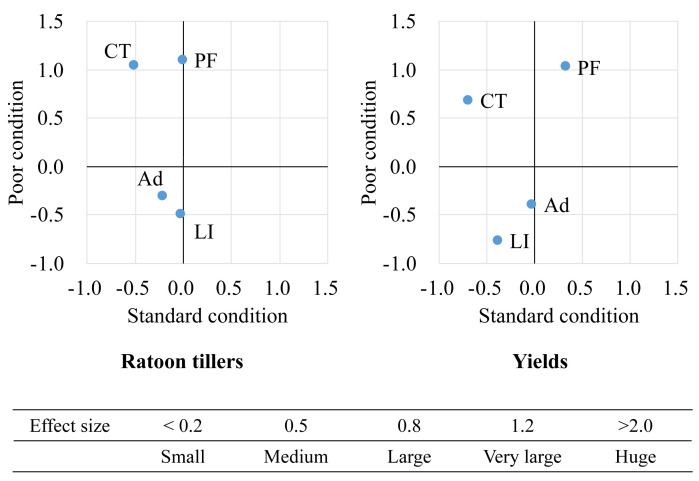
Relation of effect size between the poor and standard conditions. PF, pre-fertilization; CT, cutting twice; LI, late irrigation; Ad, Adjusting and mid-term draining. Robust practices show similar effect sizes. Unstable practices, shown by differing effect sizes, have interactions with conditions.

Effect sizes provide an evaluation involving variance and are not affected by the sample size. The effect sizes of Cohen’s *d* < 0.2, 0.5, 0.8, and 1.2, and *d* > 2.0 correspond to small, medium, large, very large, and huge, respectively ( Cohen, 1992; Sawilowsky, 2009). Figure 4 shows the relationship of the effect sizes between the conditions. The effect on ratoon tillers ( Figure 4, left) and on yield ( Figure 4, right) was similar but the effect on tillers was high under poor conditions. Pre-fertilization, cutting twice, and late irrigation had medium to large effect sizes. When the effect is near the 1:1 line, the effect is independent of the condition and is robust. A non-robust effect signifies an interaction between the practice and the conditions. Positive large effects were shown under poor conditions only.

### Reversed effect

For the effects of SALIBU management on ratoon tillers, we found an interaction between cutting twice and the cultivation conditions (standard and poor). However, the poor conditions consisted of four factors, which are as follows: low plant density, no fertilization, continuous flooding water management, and late harvesting. Therefore, which of the factors interacts with cutting twice should be clarified. The extensions of nodes by cutting twice shows the reason; although, we cannot say the meaning.

### Negative effect

Late irrigation has a robust negative effect. We can erase the negative effect by simply removing the practice. On the other hand, early irrigation may have a positive effect. In this way, an agricultural cropping method may include negative management practices if the effects are not evaluated. Our method is useful for screening positive practices in cropping methods.

### Small effect size practices

Adjusting has a robust small effect and therefore, implementation should depend on a cost-benefit analysis. In contrast, pre-fertilization has a small effect under standard conditions, but has a large effect under poor conditions. The difference shows an interaction between the practice and the condition; however, this is reasonable because the plants under poor conditions were unfertilized.

### Evaluation of cropping method

SALIBU achieved more yield than the main crop and enables the perennial cropping; however, its adaptability is unclear. Although evaluating each practice in a cropping method under different conditions is difficult because of the huge number of potential combinations, we overcame this difficulty by using an orthogonal array design pot experiment and duplicating the experiment under standard and poor conditions. Our results show that the SALIBU cropping method includes practices with unstable, negative, or small effect sizes. Improving the use of these practices could improve the method. Practices with unstable effects should be used when known to have a positive effect under a specific condition. Negative effects can be excluded by excluding the practice. Small effect practices should be used depending on the outcome of a cost-benefit analysis. Perennial ratoon rice cropping will be possible for Mekong Delta triple-cropping rice without the nine special management practices of the original SALIBU cropping method, because most of the effects of practices under standard conditions are small or negative. The triple-cropping is different with the Indonesian rice cropping; therefore, SALIBU practices should be evaluated for the Indonesian rice cropping.

## Conclusions

We examined the effect size of management practices of the SALIBU ratoon rice cropping method in triple-cropping rice in Vietnam. Cutting twice has a large effect on ratoon tillers and the effect reverses depending on the cultivation condition. Late irrigation has a robust negative effect on the yield. No additional practice is effective for perennial ratoon rice cropping in the Mekong Delta triple-cropping rice except harvesting rice near the ground because positive large effects were shown in the poor condition only. We will clarify the factors that interact with cutting twice and demonstrate ratoon cropping on fields. The use of the orthogonal array design under different conditions is useful for future studies.

## Data availability

### Underlying data

Figshare: Salibu Effect. https://doi.org/10.6084/m9.figshare.9937928.v1 ( Oda *et al.*, 2019)

Data are available under the terms of the Creative Commons Zero “No rights reserved” data waiver (CC0 1.0 Public domain dedication).
