# Supplementary material for: Evaluation of cropping method for perennial ratoon rice: Adaptation of SALIBU to triple-cropping in Vietnam
Source: F1000Res. 2020 Sep 30;8:1825. Originally published 2019 Oct 30. [Version 3] doi: 10.12688/f1000research.20890.3 (PMC7542297; doi:10.12688/f1000research.20890.3)
Supplement: Peer review history

# Peer review: Reviewer response for version 3

**DOI:** 10.5256/f1000research.29147.r72239

**Published:** 2020-10-21

**Authors:** Surajit Mondal

**Affiliations:** 1 ICAR-Research Complex for Eastern Region, Patna, Bihar, India

The authors have made the required corrections and gave explanations of the doubts raised during the review process. The article does not need further revision.

Is the work clearly and accurately presented and does it cite the current literature?

Yes

If applicable, is the statistical analysis and its interpretation appropriate?

Yes

Are all the source data underlying the results available to ensure full reproducibility?

Partly

Is the study design appropriate and is the work technically sound?

Partly

Are the conclusions drawn adequately supported by the results?

Partly

Are sufficient details of methods and analysis provided to allow replication by others?

Partly

Reviewer Expertise:

Soil science, conservation agriculture, soil organic carbon.

I confirm that I have read this submission and believe that I have an appropriate level of expertise to confirm that it is of an acceptable scientific standard.

# Peer review: Reviewer response for version 3

**DOI:** 10.5256/f1000research.29147.r72241

**Published:** 2020-10-21

**Authors:** Triadiati Antono

**Affiliations:** 1 Department of Biology, Faculty of Mathematics and Natural Sciences, IPB University, Bogor, Indonesia

Yes, I agree with the latest version.

Is the work clearly and accurately presented and does it cite the current literature?

Partly

If applicable, is the statistical analysis and its interpretation appropriate?

Partly

Are all the source data underlying the results available to ensure full reproducibility?

Yes

Is the study design appropriate and is the work technically sound?

Yes

Are the conclusions drawn adequately supported by the results?

Yes

Are sufficient details of methods and analysis provided to allow replication by others?

Partly

Reviewer Expertise:

Plant physiology and plant ecophysiology.

I confirm that I have read this submission and believe that I have an appropriate level of expertise to confirm that it is of an acceptable scientific standard.

# Peer review: Reviewer response for version 3

**DOI:** 10.5256/f1000research.29147.r72240

**Published:** 2020-10-07

**Authors:** Chenfei Dong

**Affiliations:** 1 Institute of Animal Science, Jiangsu Academy of Agricultural Sciences, Nanjing, China

No further comments.

Is the work clearly and accurately presented and does it cite the current literature?

Yes

If applicable, is the statistical analysis and its interpretation appropriate?

Yes

Are all the source data underlying the results available to ensure full reproducibility?

Partly

Is the study design appropriate and is the work technically sound?

Partly

Are the conclusions drawn adequately supported by the results?

No

Are sufficient details of methods and analysis provided to allow replication by others?

Partly

Reviewer Expertise:

My research areas are mainly on rice straw forage processing, ratoon rice cultivation and forage utilization.

I confirm that I have read this submission and believe that I have an appropriate level of expertise to confirm that it is of an acceptable scientific standard.

# Peer review: Reviewer response for version 2

**DOI:** 10.5256/f1000research.24012.r67377

**Published:** 2020-08-18

**Authors:** Surajit Mondal

**Affiliations:** 1 ICAR-Research Complex for Eastern Region, Patna, Bihar, India

The authors tried to address a noble issue with research findings. The paper is well written but has some shortcomings which are listed below:

1. The variety "Jasmine" which was used in the study was unsuitable for 'ratoon'. It will impact the result greatly. If a farmer wants to adopt ratoon system, he will select a suitable variety which is the first step of a successful production system.
2. In M&M sections, authors have not described the nine special management practices in addition to conventional management.
3. The seed rate under normal condition was 173 seeds per pot which I think give an overpopulated plant density. What is the purpose of this high seed rate?
4. For late harvesting treatment, sowing date was advanced by one week, which may actually promote the growth as well as yield.
5. There is some repetition in the results. The first and second paragraphs of results are almost the same.
6. There is a lack of data in 'Result' section and results are mostly qualitative than quantitative. Results must include the main outcomes of the research in a quantitative term. The yield is the main purpose of ratoon crop and the data related to yield is missing (except the regression graphs). A table comparing the yield under different conditions can well serve the purpose.
7. The citation pattern has a problem. The results section has the most citations while discussion section has no citations. The results section describes the main outcomes of the research while discussion section discusses the possible reasons for outcomes with supporting literature. Authors should look into the citations.

Is the work clearly and accurately presented and does it cite the current literature?

Yes

If applicable, is the statistical analysis and its interpretation appropriate?

Yes

Are all the source data underlying the results available to ensure full reproducibility?

Partly

Is the study design appropriate and is the work technically sound?

Partly

Are the conclusions drawn adequately supported by the results?

Partly

Are sufficient details of methods and analysis provided to allow replication by others?

Partly

Reviewer Expertise:

Soil science, conservation agriculture, soil organic carbon.

I confirm that I have read this submission and believe that I have an appropriate level of expertise to confirm that it is of an acceptable scientific standard, however I have significant reservations, as outlined above.

# Response

**Published:** 2020-08-26

**Authors:** Masato Oda

**Affiliations:** Japan International Research Center for Agricultural Sciences (JIRCAS), Japan

Thank you for admitting the worth of our work.

1) The variety "Jasmine" which was used in the study was unsuitable for 'ratoon'. It will impact the result greatly. If a farmer wants to adopt ratoon system, he will select a suitable variety which is the first step of a successful production system.

We think that "These disadvantages will amplify the effects of the practices; Methods-Materials L. 7". Just as using a sensitive sensor. Indeed it was. That made 0 to 0.7 tons per ha of a large range of rice yield. 

Furthermore, that is a problem if the tendency of the sensor (variety) is different from other sensors, but that point was also no matter. "The ratoon rice yield is determined by the number of ratoon tillers, and the relationship between the number of ratoon tillers and the yield is consistent with those reported in a previous study (Pasaribu et al., 2018); Result-Yield component L. 3".

2) In M&M sections, authors have not described the nine special management practices in addition to conventional management.

That is in the Methods-Treatments.

3) The seed rate under normal condition was 173 seeds per pot which I think give an overpopulated plant density. What is the purpose of this high seed rate?

That is the standard in in the Mekong Delta. The aim of this work is adaptation of SALIBU to triple-cropping in Vietnam. 

4) For late harvesting treatment, sowing date was advanced by one week, which may actually promote the growth as well as yield.

That is matter of the main crop. The ratoon cropping started at the same day and harvested the same day. 

5) There is some repetition in the results. The first and second paragraphs of results are almost the same.

That is a style for readability for average readers. You have a good memory than ordinary people if you feel it redundant. 

6) There is a lack of data in 'Result' section and results are mostly qualitative than quantitative. Results must include the main outcomes of the research in a quantitative term. The yield is the main purpose of ratoon crop and the data related to yield is missing (except the regression graphs). A table comparing the yield under different conditions can well serve the purpose.

You can see the table by clicking the URL in Underlying data section.  

7) The citation pattern has a problem. The results section has the most citations while discussion section has no citations. The results section describes the main outcomes of the research while discussion section discusses the possible reasons for outcomes with supporting literature. Authors should look into the citations.

We agree with you; however, there are few papers in English for SALIBU.  In addition, our work is the first work of adaptation of SALIBU to triple-cropping in Vietnam, probably. The statistical analysis of the cropping methods component is also very rare. The discussion section was combined with the results section.

# Peer review: Reviewer response for version 2

**DOI:** 10.5256/f1000research.24012.r61311

**Published:** 2020-03-25

**Authors:** Chenfei Dong

**Affiliations:** 1 Institute of Animal Science, Jiangsu Academy of Agricultural Sciences, Nanjing, China

The research point is very interesting and worthy of affirmation. However, I think there are several problems in the design of this experiment. First, the rice variety adopted in this experiment is not suitable for ratoon use, so the results could not explain the impact of SALIBU  measures on ratoon rice well. Second, the soil fertility of standard conditions and the climatic factors such as sun light and temperature of the experiment site are not explained, so it is difficult to compare the results with previous reports. What’s more, there is no introduction for the fertilizer status of the tested soil, so it is difficult to discuss the changes of soil under four adverse environments, which is the important reason that leads to the conclusion of the study not being convincing.

Is the work clearly and accurately presented and does it cite the current literature?

Yes

If applicable, is the statistical analysis and its interpretation appropriate?

Yes

Are all the source data underlying the results available to ensure full reproducibility?

Partly

Is the study design appropriate and is the work technically sound?

Partly

Are the conclusions drawn adequately supported by the results?

No

Are sufficient details of methods and analysis provided to allow replication by others?

Partly

Reviewer Expertise:

My research areas are mainly on rice straw forage processing, ratoon rice cultivation and forage utilization

I confirm that I have read this submission and believe that I have an appropriate level of expertise to confirm that it is of an acceptable scientific standard, however I have significant reservations, as outlined above.

# Response

**Published:** 2020-04-23

**Authors:** Masato Oda

**Affiliations:** Japan International Research Center for Agricultural Sciences (JIRCAS), Japan

Thank you for admitting the worth of our work.

**First, the rice variety adopted in this experiment is not suitable for ratoon use, so the results could not explain the impact of SALIBU  measures on ratoon rice well.**

We think that "These disadvantages will amplify the effects of the practices; Methods-Materials L. 7". Just as using a sensitive sensor. Indeed it was. That made 0 to 0.7 tons per ha of a large range of rice yield. 

Furthermore, that is a problem if the tendency of the sensor (variety) is different from other sensors, but that point was also no matter. "The ratoon rice yield is determined by the number of ratoon tillers, and the relationship between the number of ratoon tillers and the yield is consistent with those reported in a previous study (Pasaribu et al., 2018); Result-Yield component L. 3".

**Second, the soil fertility of standard conditions and the climatic factors such as sun light and temperature of the experiment site are not explained, so it is difficult to compare the results with previous reports.**

The aim of this study is to adopt SALIBU practices for the triple-rice cultivation in the Mekong Delta in Vietnam. This area is in the same climate area and most of the soil is the same. 

More essentially, the orthogonal array design experiment under large noise established by Taguchi (1986) enables the utilization of the practice having a robust effect found by the experiment to the actual field. Therefore, we expect that our results are robust for other tropical countries. Actually, our colleague belonging to another project recognized (unpublished) the same result in Myanmar. We recommend you to read the Taguchi's paper because the way of thinking is very useful.

**What’s more, there is no introduction for the fertilizer status of the tested soil, so it is difficult to discuss the changes of soil under four adverse environments, which is the important reason that leads to the conclusion of the study not being convincing.**

What do you mean the "status"? Do you mean soil nutrition properties? We think soil analysis does no effect on the conclusion of this experiment because with and without fertilizing made a large difference. Our point of view is the evaluation of the robustness and size of the effects of practices under large noises.

We are waiting for your response.

# Response

**Published:** 2020-08-26

**Authors:** Masato Oda

**Affiliations:** Japan International Research Center for Agricultural Sciences (JIRCAS), Japan

We added the explanation for the Taguchi method as follows.

"The Taguchi method is a popular method to test the robustness of technologies in actual condition by artificial condition."

# Peer review: Reviewer response for version 2

**DOI:** 10.5256/f1000research.24012.r57979

**Published:** 2020-01-20

**Authors:** Triadiati Antono

**Affiliations:** 1 Department of Biology, Faculty of Mathematics and Natural Sciences, IPB University, Bogor, Indonesia

I have read the revision version of the article. If the results still cite the references, while the discussion does not cite the references, it is recommended that the results and discussion be combined.

Is the work clearly and accurately presented and does it cite the current literature?

Partly

If applicable, is the statistical analysis and its interpretation appropriate?

Partly

Are all the source data underlying the results available to ensure full reproducibility?

Yes

Is the study design appropriate and is the work technically sound?

Yes

Are the conclusions drawn adequately supported by the results?

Yes

Are sufficient details of methods and analysis provided to allow replication by others?

Partly

Reviewer Expertise:

Plant physiology and plant ecophysiology.

I confirm that I have read this submission and believe that I have an appropriate level of expertise to confirm that it is of an acceptable scientific standard, however I have significant reservations, as outlined above.

# Response

**Published:** 2020-01-23

**Authors:** Masato Oda

**Affiliations:** Japan International Research Center for Agricultural Sciences (JIRCAS), Japan

For the first, let me express my heartfelt appreciation again to you for finding the mistake of the reference.

However, we cannot accept to combine the results and discussion because of the guideline of F1000Research (Results section must be independent, Conclusion and Discussion can combine). 

We are using the reference to indicate that our result is consistent with the previous study (the relation between the yield and the tillers). That is "what our result is", not "what our result means". We should describe "what our results are" in the result section and "what our results mean" in the discussion section. In particular, the aim of our study is the evaluation of management practices of SABIBU in triple-rice cropping. The referenced paper is valuable but that is for SRI on SALIBU. We described triple-rice cropping in the discussion section.

I think authors should follow the guidelines of F1000Research, and I hope you accept our idea.

# Response

**Published:** 2020-08-26

**Authors:** Masato Oda

**Affiliations:** Japan International Research Center for Agricultural Sciences (JIRCAS), Japan

Finally, we could have been get a suggestion from editorial team to obey your recommendation. We combined the results and discussion.

# Peer review: Reviewer response for version 1

**DOI:** 10.5256/f1000research.22984.r56890

**Published:** 2019-12-09

**Authors:** Le Thi Hoa Sen

**Affiliations:** 1 Hue University of Agriculture and Forestry, Hue, Vietnam

The abstract well conveys the research objectives, methods, key research results, discussion and conclusions. However, it needs to be shorter and more precise. 

 The study found very significant results for rice producers in three-crop rice-producing areas or in lowland areas that are vulnerable to natural disasters. However, the results will be more convincing if the collected data is analyzed more deeply, concerning the causes of fluctuating results. For instance, under poor conditions the effect of cutting twice was positive but negative under standard condition, why? What might be the causes? It needs further analyses of the results.

 In addition, to be more practical and to validate research findings, experiments should be carried out one more time in the net house or in the real conditions.

Is the work clearly and accurately presented and does it cite the current literature?

Yes

If applicable, is the statistical analysis and its interpretation appropriate?

Partly

Are all the source data underlying the results available to ensure full reproducibility?

Yes

Is the study design appropriate and is the work technically sound?

Yes

Are the conclusions drawn adequately supported by the results?

Yes

Are sufficient details of methods and analysis provided to allow replication by others?

Partly

Reviewer Expertise:

Adaptation to climate change in agriculture production.

I confirm that I have read this submission and believe that I have an appropriate level of expertise to confirm that it is of an acceptable scientific standard, however I have significant reservations, as outlined above.

# Response

**Published:** 2019-12-16

**Authors:** Masato Oda

**Affiliations:** Japan International Research Center for Agricultural Sciences (JIRCAS), Japan

Thank you for your helpful comments. We shortened the abstract and made it more precise. We also changed the title too.

About the cutting twice, we are conducting an additional experiment. That is a portion of developing SALIBU for Mekong Delta triple cropping. We started the field experiment with suitable varieties.

# Peer review: Reviewer response for version 1

**DOI:** 10.5256/f1000research.22984.r55968

**Published:** 2019-12-02

**Authors:** Triadiati Antono

**Affiliations:** 1 Department of Biology, Faculty of Mathematics and Natural Sciences, IPB University, Bogor, Indonesia

1. The work is not yet clearly and accurately presented, nor does it cite the current literature. The literature review is limited, especially about salibu (definition, advantages, yield). The problems are not clear yet. What is the importance of this research (please use Pertanika *et al.,* 2018 ^1^ as reference for example, because the article states the advantages.)
2. Because the methods (the treatments) are not clear and analysis does not use tools, the methods are not provided to allow replication by others.
3. Because the statistical analysis does not explain the tools used, it will be difficult to interpret easily
4. Results: did not need to state references.
5. Results: please just write down the results of the study, without mentioning the references.
6. Fig 1-3, Tab 1: please use statistical analysis to explain the results.
7. Discussion: explain the reasons for the results, use the references to discuss and compare research results. Please, use references for discussion. 

Is the work clearly and accurately presented and does it cite the current literature?

Partly

If applicable, is the statistical analysis and its interpretation appropriate?

Partly

Are all the source data underlying the results available to ensure full reproducibility?

Yes

Is the study design appropriate and is the work technically sound?

Yes

Are the conclusions drawn adequately supported by the results?

Yes

Are sufficient details of methods and analysis provided to allow replication by others?

Partly

Reviewer Expertise:

Plant physiology and plant ecophysiology.

I confirm that I have read this submission and believe that I have an appropriate level of expertise to confirm that it is of an acceptable scientific standard, however I have significant reservations, as outlined above.

## References

[1] : Rice Ratooning Using the Salibu System and the System of RiceIntensification Method Influenced by Physiological Traits. *Pertanika J. Trop. Agric. Sci.*.2018;41(2) :637-654

# Response

**Published:** 2019-12-16

**Authors:** Masato Oda

**Affiliations:** Japan International Research Center for Agricultural Sciences (JIRCAS), Japan

1. Thank you for your helpful comments. Our work has two aspects, evaluating SALIBU method, and adopting SAIBU to Mekong Delta Triple cropping. The former is means and the latter is the aim. We changed the title and improved the abstract and introduction.
2. Thank you so much for introducing the correct citation. We referred the paper but mistakenly typed another paper wrote by the same authors in the same year. We corrected that. Thanks again.
3. We guess that you mention about a table of the treatments. That is provided in "figshare". We added the link to the methods section. Please kindly point specifically if that is not enough.
4. For the tools, We show "Microsoft Excel 2016" and "the formula of the Cohens’ d effect size". That is enough to replicate our work. We provided the raw data by "figshare" and show the link. This is the regulation of F1000Research.
5. For the location of citation, F1000Research has no regulations. Citations in the result section are commonly seen. For example, a famous book, “Science Research Writing: A Guide for Non-Native Speakers of English”, recommends no references should use unless essential in the discussion section.
6. We added a statistical analysis to Figures 1-3 and Table 1.
